# *Brucella* Control of Dendritic Cell Maturation Is Dependent on the TIR-Containing Protein Btp1

**DOI:** 10.1371/journal.ppat.0040021

**Published:** 2008-02-08

**Authors:** Suzana P Salcedo, María Ines Marchesini, Hugues Lelouard, Emilie Fugier, Gilles Jolly, Stephanie Balor, Alexandre Muller, Nicolas Lapaque, Olivier Demaria, Lena Alexopoulou, Diego J Comerci, Rodolfo A Ugalde, Philippe Pierre, Jean-Pierre Gorvel

**Affiliations:** 1 Centre d'Immunologie de Marseille-Luminy, Aix Marseille Université, Faculté de Sciences de Luminy, Marseille, France; 2 INSERM, U631, Marseille, France; 3 CNRS, UMR6102, Marseille, France; 4 Instituto de Investigaciones Biotecnológicas, IIB-INTECH Universidad Nacional de San Martin, San Martin, Buenos Aires, Argentina; 5 Consejo Nacional de Investigaciones Cientificas y Tecnologicas CONICET-INTI-Ed. 24-, San Martin, Buenos Aires, Argentina; Duke University Medical Center, United States of America

## Abstract

*Brucella* is an intracellular pathogen able to persist for long periods of time within the host and establish a chronic disease. We show that soon after *Brucella* inoculation in intestinal loops, dendritic cells from ileal Peyer's patches become infected and constitute a cell target for this pathogen. In vitro, we found that *Brucella* replicates within dendritic cells and hinders their functional activation. In addition, we identified a new *Brucella* protein Btp1, which down-modulates maturation of infected dendritic cells by interfering with the TLR2 signaling pathway. These results show that intracellular *Brucella* is able to control dendritic cell function, which may have important consequences in the development of chronic brucellosis.

## Author Summary

A key determinant for intracellular pathogenic bacteria to induce infectious diseases is their ability to avoid recognition by the host immune system. Although most microorganisms internalized by host cells are efficiently cleared, *Brucella* behave as a Trojan horse causing a zoonosis called brucellosis that affects both humans and animals. Here we show that pathogenic *Brucella* are able to target host cell defense mechanisms by controlling the function of the sentinels of the immune system, the dendritic cells. In particular, the *Brucella* TIR-containing protein (Btp1) targets the Toll-like receptor 2 activation pathway, which is a major host response system involved in bacterial recognition. Btp1 is involved in the inhibition of dendritic cell maturation. The direct consequence is a control of inflammatory cytokine secretion and antigen presentation to T lymphocytes. These bacterial proteins are not specific for *Brucella* and have been identified in other pathogens and may be part of a general virulence mechanism used by several intracellular pathogens to induce disease.

## Introduction

The immune response to bacterial infection relies on the combined action of both the innate and adaptive immune systems. Dendritic cells (DCs) are known as mediators of pathogen recognition and are strategically located at the typical sites of bacterial entry and have the ability to migrate from peripheral tissues to secondary lymphoid organs to elicit primary T cell responses and initiate immunity.

DCs express several pathogen recognition receptors such as C-type lectins and Toll-like receptors (TLRs) that recognise molecular patterns expressed by pathogens and determine the type of immune response. Microbial stimuli induce significant morphological and biochemical changes, such as IL-12 secretion and increased surface expression of many co-stimulatory and MHC class II molecules. This activation of DCs, known as maturation, is required for efficient T-cell-priming and pathogen elimination. However, several bacterial pathogens have developed mechanisms to subvert DC function and evade the immune system. Mycobacterium tuberculosis interferes with TLR signalling via the C-type lectin DC-SIGN, blocking DC maturation and IL-12 production [1]. The immune response is instead directed towards immune suppression with secretion of IL-10, which seems to contribute to the chronic carriage of this pathogen. Similarly, Bordetella pertussis-infected DCs secrete IL-10 and activate T regulatory cells that suppress a protective immune response and enhance colonisation of the lower respiratory tract [2]. In addition, several studies have shown that while Salmonella typhimurium induces normal maturation of DCs and secretion of pro-inflammatory cytokines [3,4] it is able to block MHC class II antigen presentation in bone marrow-derived DCs [5,6]. Another bacterial pathogen, Francisella tularensis, which also induces phenotypic maturation of DCs, has been shown to inhibit secretion of pro-inflamatory cytokines such as TNF-α while eliciting production of immunosuppressive cytokines that facilitate pulmonary infection [7]. F. tularensis has also been shown to replicate efficiently within DCs [7] in contrast to many other bacterial pathogens (including mycobacteria, *Bordetella*, *Salmonella* and Legionella pneumophila) for which DCs seem to restrict their intracellular growth.

Human brucellosis is a zoonotic disease that results from direct contact with infected animals or ingestion of contaminated food products. It is usually presented as a debilitating febrile illness that can progress into a chronic disease with severe complications such as infection of the heart and bones. It has been previously shown that B. abortus infection in calves using an intestinal loop model occurred via Peyer's patches [8]. Several studies using animal models have established macrophages and placental trophoblasts as two key targets of *Brucella* infection [9] but a more recent study has shown that *Brucella* can replicate *in vitro* within human monocyte-derived DCs [10]. DCs may therefore constitute an important cellular niche to promote infection.

*Brucella* virulence is dependent on its ability to survive and replicate within host cells. Once internalised, *Brucella* is found within a compartment, the *Brucella*-containing vacuole (BCV) that transiently interacts with early endosomes [11]. However, BCVs do not fuse with lysosomes and instead fuse with membrane from the endoplasmic reticulum (ER), to establish a vacuole suited for replication [11,12]. An important virulence factor of *Brucella* is its unconventional lipopolysaccharide (LPS) that is necessary for entry and early development of BCVs within host cells [13]. *Brucella* LPS also has important immuno-modulatory activities by forming non-functional complexes with MHC class II at the cell surface of macrophages [14]. More recently, the periplasmic *Brucella* cyclic β-1,2-glucan, which interacts with lipid domains, has been implicated in the early biogenesis of BCVs [15]. At later stages of BCV trafficking, the VirB type IV secretion system enables interactions with the ER and formation of an ER-derived compartment suitable for *Brucella* intracellular replication [12,16,17]. However, no secreted effector molecules of the VirB type IV secretion system have yet been identified.

Although there is increasing knowledge about the virulence factors that enable *Brucella* to survive inside host cells, it is still unclear how *Brucella* is able to remain hidden from the immune system and cause chronic disease. The escape from the lysosomal pathway and fusion with the ER may interfere with the ability of host cells to mount an efficient immune response against *Brucella.* In this study, we used a murine intestinal loop model of infection and found that intestinal DCs from the region underlying the follicle associated epithelium (FAE) of Peyer's patches were infected by B. abortus soon after inoculation. To better characterise the consequences of B. abortus infection on the immune functions of DCs, we have used murine bone marrow-derived dendritic cells (BMDCs) as a model system. B. abortus replicates within BMDCs and inhibits the process of DC maturation, ultimately leading to a reduction of cytokine secretion and antigen presentation. We have also identified a *Brucella* protein, Btp1, which contributes to inhibiting maturation of infected DCs *in vitro* by targeting the TLR2 signalling pathway, highlighting a new mechanism used by *Brucella* to control the immune response of the host.

## Results

### B. abortus Infection of Intestinal DCs

**Figure 1:**
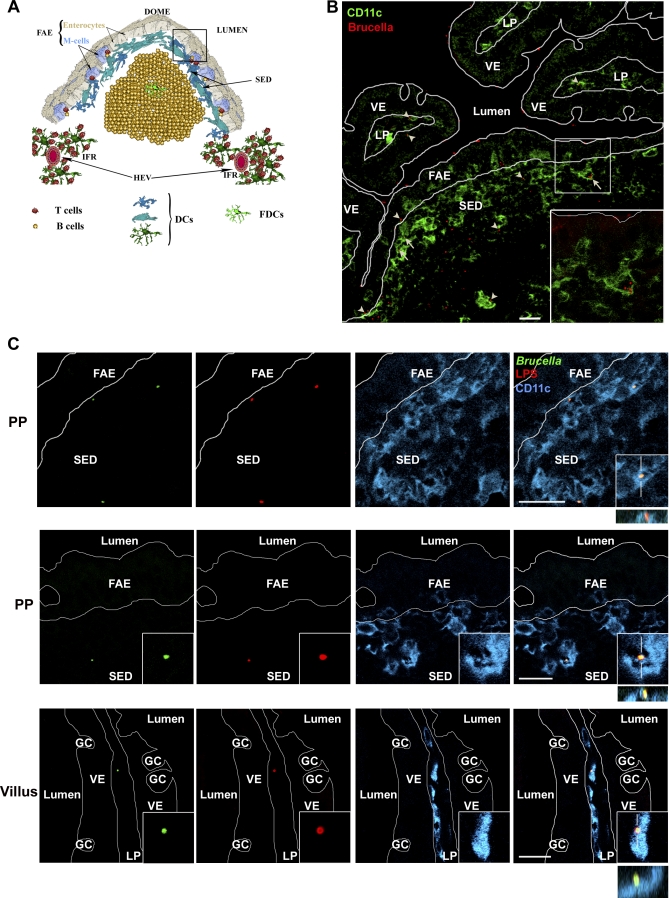
Infection of DCs from the Sub-epithelial Dome Region of Ileal Peyer's Patches with B. abortus (A) Cellular organization of a Peyer's patch dome. DCs are enriched in the Sub-Epithelial Dome (SED) underlying the Follicle-Associated Epithelium (FAE) and in the Inter-Follicular Region (IFR) in vicinity of the High Endothelial Venules (HEV). (B) Sixty minutes after intestinal loop infection, B. abortus (red) adhered on both villi epithelium (VE) and FAE surfaces. However, only few of them were observed in villi lamina propria (LP). These were always observed in association with CD11c+ cells (green, arrowhead). Most B. abortus bacteria reached the under-epithelial compartment of the gut through the FAE. Some of these were observed inside CD11c+ cells (arrows and insert), while others were in close association with CD11c+ dendrites (arrowheads). (C) High magnification field of a Peyer's patch infected with GFP-expressing *Brucella*. Images in the first and the second row, corresponding to area depicted by the squared box in (A), show either *Brucella* (GFP in green and LPS in red) internalized by CD11+ cells (blue, representative of 29 out of 118 B. abortus-associated cells) or the close association of one *B. abortus* expressing GFP with dendrites from a CD11c+ cell of the SED region (representative of 30 out of 118 B. abortus-associated cells), respectively. The image in the last row shows one of the rare B. abortus-associated cells (all CD11c+) from the villus (GC, goblet cells). Close association or internalization was always confirmed by the combined xy, xz, and/or yz views as shown in inserts. Bars, 20 μm.

We used a mouse intestinal loop model to characterise the cellular targets of B. abortus by confocal microscopy during early stages of the infection. One hour after inoculation in a loop containing at least one Peyer's patch, B. abortus mainly penetrated the epithelium through the FAE of Peyer's patches and were either associated or internalized by cells presenting a “dendritic-like” morphology and identified as DCs on the basis of their positivity for CD11c (Figure 1). On more than one hundred cells associated with B. abortus we observed, 50% were CD11c+ cells, the others being either FAE cells (30%) or not determined. Few bacteria were also observed in the lamina propria of adjacent villi (Figure 1). These were always associated with CD11c+ cells. Thus, in the mouse model, DCs are a cellular target of B. abortus during enteric infection.

### B. abortus Replicates within ER-derived Vacuoles in Murine BMDCs

**Figure 2:**
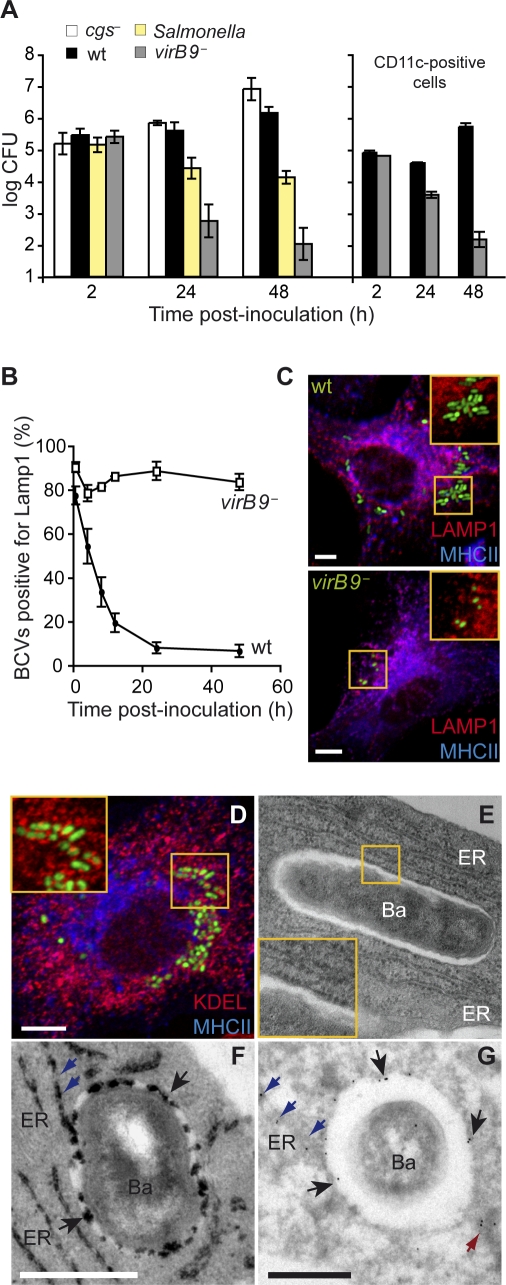
Survival and Replication of B. abortus in DCs (A) BMDCs infected with wild-type B. abortus, *cgs* ^−^ mutant, *virB9* ^−^ mutant or the wild-type S. typhimurium were lysed and intracellular CFUs enumerated at different times after inoculation. Graph on the right corresponds to bacterial CFU counts performed in CD11c-positive cells infected with either wild-type B. abortus or *virB9* ^−^ mutant. Statistical significance was obtained at 24 and 48 h between wild-type and the *virB* mutant (*p* < 0.002) and also between wild-type *Brucella* and *Salmonella* at 24 h (*p* = 0.047) and 48 h (*p* < 0.002). (B) Quantification of the percentage of wild-type or *virB9* ^−^ mutant BCVs that contain LAMP1 by confocal immunofluorescence microscopy. The difference between wild-type and the *virB* mutant were always statistically significant (*p* = 0.045 at 30 min and 4 h; *p* < 0.002 for other timepoints). (C) Representative confocal images of DCs infected for 24 h with wild-type B. abortus or *virB9* ^−^ mutant expressing GFP, labelled with antibodies against MHC II (blue) and LAMP1 (red). (D) Confocal image of DCs infected for 24 h with wild-type B. abortus expressing GFP, labelled with antibodies against MHC II (blue) and KDEL (red). Samples were also processed for conventional electron microscopy (E), cytochemistry for glucose 6 phosphatase detection (F) or immunogold labelling with an anti-calnexin antibody (G). Bars: 5 μm (C and D); 0.5 μm (F and G). For (A) and (B) data are means ± standard errors of four independent experiments.

Having established the relevance of DCs in the context of *Brucella* infection we used BMDCs as cellular model to characterise the intracellular fate of *Brucella*. We first analysed the survival of the wild-type B. abortus 2308 strain in murine BMDCs by enumerating the colony forming units (CFU) at different times after infection. We observed an increase in the number of intracellular bacteria up to 48 h after inoculation, showing that the wild-type B. abortus is able to survive within BMDCs (Figure 2A). Equivalent results were obtained in CD11c-positive cells (Figure 2A, graph on the right). Intracellular replication was confirmed by microscopic examination of infected cells. At 2 h after infection cells contained on average 1.2 ± 0.1 bacteria per cell in contrast to 17.7 ± 3.4 bacteria per cell at 48h. Although high intracellular bacterial loads were observed, viability of BMDCs was not affected up to 48 h after infection (Figure S1). At 24 and 48 h we found that only a small percentage of infected cells (approximately 12 and 25%, respectively) contained more than 10 bacteria, indicative of intracellular replication. This observation is similar to what was previously described for murine bone marrow-derived macrophages [12] and HeLa cells [11]. In agreement with previous reports, we did not observe replication with the wild-type Salmonella typhimurium [18]. In the case of a strain lacking the type IV secretion component *virB9*, there was a continuous decrease in CFU numbers, suggesting that this system is necessary for the establishment of the *Brucella* replicative niche in DCs. Surprisingly, in contrast to other cell types [15,19] the cyclic β-1,2 glucan synthesized by *Brucella* was not required for virulence during DC infection since *cgs* ^−^ mutants replicated as efficiently as the wild-type strain (Figure 2A).

Loss of lysosomal and acquisition of ER markers on BCVs are hallmarks of *Brucella* infection. We therefore analysed the recruitment of lysosomal associated membrane protein 1 (LAMP1) and of the ER-specific KDEL bearing molecules in MHC II expressing CD11c-positive DCs by immunofluorescence confocal microscopy (Figures 2 and S2). As expected, *virB9* ^−^ mutant BCVs retained LAMP1 whereas wild-type BCVs progressively lost this marker (Figure 2B). At 24 h after infection only a small percentage of wild-type BCVs contained LAMP1 in contrast to the *virB9* ^−^ mutant (Figure 2C). Concomitant to LAMP1 decrease, a significant proportion of wild-type BCVs became positive for the ER retention and retrieval epitope KDEL (50.7 ± 3.5%, Figure 2D), albeit to a lower level than in macrophages [12]. Wild-type BCV fusion with ER membranes was confirmed through ultrastructural examination by electron microscopy of infected DCs. BCVs were often outlined by ribosomes (Figure 2E) and cytochemical detection of glucose 6-phosphatase activity, an enzyme mostly found in the ER lumen, revealed that the majority of BCVs were positive for this enzymatic activity at 24 h after infection. In cryosections of infected DCs we observed ER membranes directly fusing with BCVs (Figure 2F, blue arrows), which were also decorated with an antibody against the ER resident protein calnexin (Figure 2G). In addition to the ER cisternae, which were extensively labelled (data not shown), gold particles were present on the vacuolar membranes surrounding *Brucella* and on tubular-like ER membranes connecting with the vacuoles (blue arrows) as well as on vesicles in the vicinity of BCVs (red arrow). Together, these results confirm that *Brucella* is dependent on its VirB type IV secretion system to replicate within DCs in an ER-derived compartment that evades the lysosomal degradative pathway, similar to what has been observed for other cell types, in particular within bone marrow-derived macrophages [12].

### B. abortus Interferes with Phenotypic Maturation of DCs

**Figure 3:**
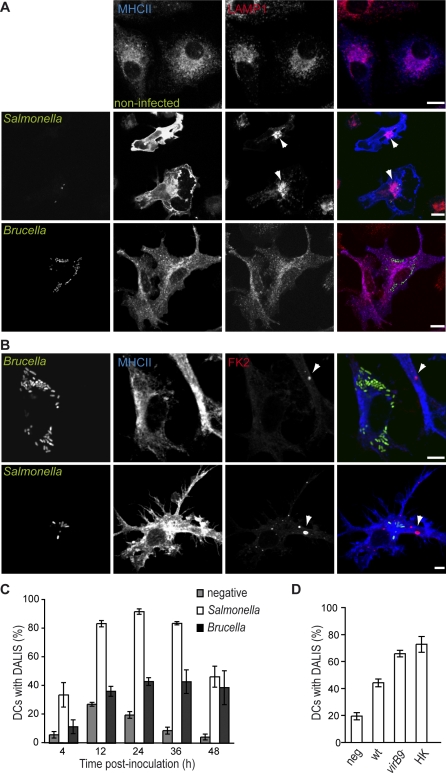
B. abortus Interferes with DC Maturation DCs were infected with different B. abortus strains or with *Salmonella* and labelled with the anti-MHC II and anti-LAMP1 antibodies (A) or FK2 antibody (B) to detect MVBs or DALIS, respectively. (C) Comparison between the percentages of DCs infected with either wild-type B. abortus (black bars) or S. typhimurium (white bars) that contain DALIS. The negative control (grey bars) corresponds to mock-infected DCs with DALIS. The difference between *Brucella* and *Salmonella* infected cells at 24 h is statistically significant, *p* < 0.0001. (D) Quantification of the percentage of DCs with DALIS at 24 h after incubation with media (negative), wild-type B. abortus (wt), *virB9* ^−^ mutant or heat-killed B. abortus (HK). The differences between wild-type wt and either *virB9* ^−^ or HK are statistically significant (*p* = 0.0059 and *p* = 0.018, respectively). For (C) and (D) data represent means ± standard errors of at least three independent experiments. Bars: 10 μm (A); 5 μm (B).

Most studies have shown that infection of DC is generally associated with their activation and a mature phenotype. This phenotype is characterized not solely by the up-regulation of co-stimulatory and MHC class II molecules at the cell surface, but also by the intense clustering of multivesicular bodies (MVB) around the microtubule organizing center (MTOC) [20], as well as the formation of large cytosolic dendritic cell aggresome-like induced structures (DALIS), which are made of defective newly synthesised ubiquitinated proteins [21]. This situation is well illustrated by *Salmonella*-infected DCs (Figure 3A), in which MHC class II molecules mostly localize on the cell surface and MVBs, labelled with LAMP1, are often tightly concentrated at the MTOC*.* Surprisingly, most of *Brucella*-infected DCs did not show any sign of maturation, since *Brucella*-bearing cells rarely displayed lysosomal clustering or MHC II surface accumulation (Figure 3A). Instead, MHC II molecules remained mostly intracellular co-localising with LAMP1 as observed in non-activated DCs. At 24 h after inoculation, only 14.3 ± 0.5% of *Brucella-*infected DCs (versus 31.9 ± 8.3% for *Salmonella*) displayed a mature phenotype in terms of MHC II molecules and LAMP1 distribution, suggesting that *Brucella* does not promote DC activation.

We then tested if DALIS formation was also impaired in infected cells. Although the function of DALIS is still unclear, these structures are easily detectable using the FK2 antibody that recognises both mono- and polyubiquitinated proteins, and therefore provide an effective way of monitoring DC maturation by microscopy. Thus, we compared the kinetics of DALIS formation in DCs infected with either *Brucella* or *Salmonella*, previously shown to induce normal maturation of DCs [3,6] (Figure 3B–3D). In the case of *Salmonella*, formation of DALIS began after 4 h (as reported with E. coli LPS [21]), with 90% of infected cells containing large and numerous DALIS 24 h after infection (Figure 3B and 3C), while only 20% of control cells contained DALIS, probably due to mechanical or spontaneous maturation. Although significantly higher than the control (*p* = 0.005), only 43% of *Brucella*-infected DCs contained DALIS 24 h after infection and this number remained stable a later time-points (Figure 3C). Furthermore, the average size of *Brucella*-induced DALIS was always considerably smaller than that of DALIS formed in the presence of *Salmonella* (Figure 3B). These observations suggest the *Brucella* replication in DC could be restricting the maturation process. A significantly higher proportion of DCs infected with *virB9* ^−^, a mutant that does not replicate in DCs*,* contained DALIS at 24 h after infection (*p* = 0.006, Figure 3D). Similar results were obtained when analysing DCs activated with heat-killed B. abortus (*p* = 0.018, Figure 3D), consistent with the hypothesis that B. abortus is actively inhibiting the maturation of DCs.

**Figure 4:**
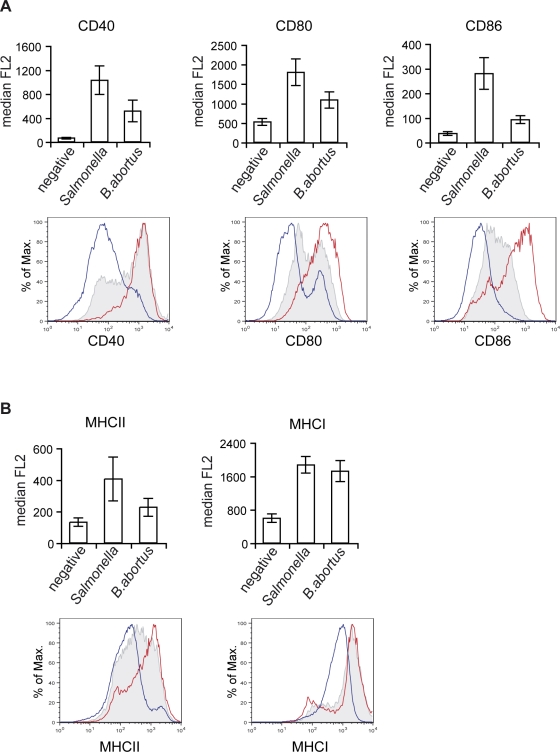
B. abortus-infected DCs Have Reduced Expression of Co-stimulatory Molecules (A and B) DCs were either incubated with media only (blue line) or infected with B. abortus (shaded grey) or S. typhimurium (red line) expressing GFP for 24 h. DCs were labelled with anti-CD11c conjugated with APC and anti-CD40, CD80, CD86, MHC II, and MHCI antibodies conjugated with PE. Representative histograms and values of median of the PE fluorescence (FL2) correspond to CD11c^+^ cells for the negative and CD11c^+^ GFP^+^ cells for the *Brucella* and *Salmonella* infected cells. Values of the median fluorescence correspond to four independent experiments.

The extent of this inhibition was monitored by flow cytometry of CD11c-positive DCs infected with GFP-expressing *Brucella* or *Salmonella*, for surface expression of classical maturation markers (eg CD80, CD86 and MHC molecules). Analysis of the median of fluorescence (Figure 4A) or the geometric mean (data not shown) in the CD11c^+^GFP^+^ populations showed a consistently lower surface expression of all co-stimulatory and MHC class II molecules in cells infected with B. abortus than in *Salmonella*-infected cells (Figure 4A and 4B). However, statistical difference between B. abortus and *Salmonella* was only observed for CD86 expression (*p* = 0.011). This is probably due to the fact that in the case of *Brucella-*infected DCs two populations were observed (overlay histograms, Figure 4A and 4B), one with a moderately increased surface levels in comparison to control cells and the other with similar levels of expression to *Salmonella*-activated DCs. This is in agreement with microscopy observations where a high proportion of MHC class II molecules remained intracellular in *Brucella*-infected cells (Figure 2A). Surprisingly, we did not detect any significant difference in the surface expression of MHC class I molecules in cells infected by the two pathogens (Figure 4B), thus suggesting the expression of MHC class I molecules is controlled differently than MHC class II and co-stimulatory molecules. Together, these data confirm that maturation of *Brucella*-infected DCs is impaired.

In addition, we have found that *Brucella* can only establish its intracellular replicative niche in immature DCs (Figure S3). When E. coli LPS was added to cells (to induce maturation) early after infection (0.5 to 6 h), there was a significant inhibition of bacterial growth (*p* < 0.001, Figure S3). However, bacterial CFU counts did not decrease when LPS was added at 12 h after infection, suggesting that *Brucella* survival is not affected by DC maturation at a time point that corresponds to its arrival in the ER [11,12]. In contrast, addition of E. coli LPS had no significant effect on the intracellular survival of *Salmonella* in DCs (data not shown), thus further underlining the need for *Brucella* to avoid DC maturation.

### *Brucella* Interferes with DC Function

**Figure 5:**
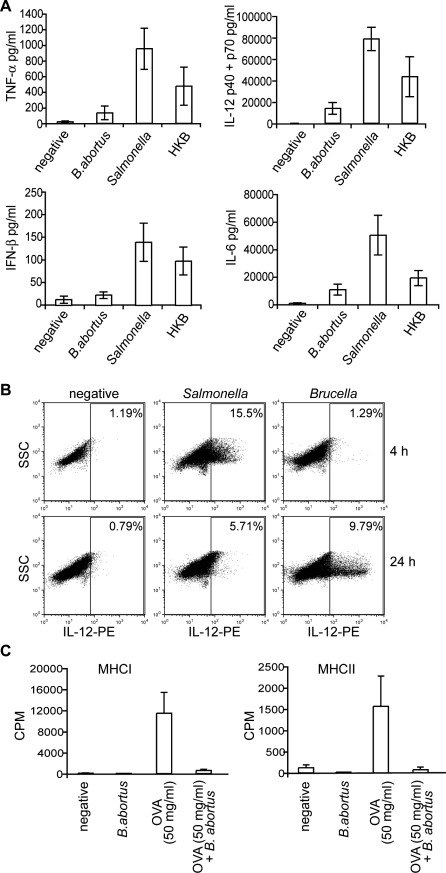
B. abortus Interferes with DC Function (A) and (B) B. abortus blocks secretion of pro-inflammatory cytokines. (A) Supernatants were obtained at 24 h from DCs inoculated with either media (negative), B. abortus, S. typhimurium, or heat-killed B. abortus (HKB) and the levels of cytokines were determined by ELISA. Values correspond to means ± standard errors of at least three independent experiments. A clear statistical difference was observed between *Brucella* and *Salmonella* for TNF (*p* = 0.014) and IL12 (*p* = 0.0098) and to a lesser extent for IL6 (*p* = 0.041) and IFNß (*p* = 0.040). (B) DCs were infected for 4 or 24 h with either media (negative), B. abortus or S. typhimurium constitutively expressing GFP. DCs were then treated for 1 h with brefeldin A, permeabilised and labelled with anti-CD11c and anti-IL12 (p40/p70) conjugated with APC and PE, respectively. Representative dot blots are shown for CD11c^+^ populations. (C) B. abortus reduces the capability of DCs to induce T cell proliferation. DCs were infected with wild-type B. abortus and the model antigen ovabulmin (OVA) was added after 30 min at a final concentration of 50 μg/ml. Cells were stimulated with OVA for 2 h and then washed to remove the antigen. Splenic CD4^+^ T cells prepared from OTI and OTII transgenic mice were then added at a 1:1 ratio and co-cultured for 48 h. IL-2 production in culture supernatants was measured by ^3^H-thymidine incorporation on the IL-2-dependent CTLL-2 cell line. Data are the means ± standard errors from triplicates of a representative experiment. A small statistical difference was observed between Ova and Ova+*Brucella* for both OT1 (*p* = 0.035) and OT2 (*p* = 0.045).

DCs infected with B. abortus do not achieve full maturation. We therefore investigated the consequences of *Brucella* infection on the ability of DCs to secrete cytokines, by analysing the supernatants of infected cells. Levels of TNF-α, IL-12 (p40/p70), IL-6 and IFN-β were consistently and considerably lower in supernatants from *Brucella*- than from *Salmonella*-infected cells (Figure 5A). This effect was observed at 24 h as well as at 48 h after infection (data not shown) suggesting that *Brucella* infection inhibits the secretion of immuno-stimulating cytokines.

Intracellular IL-12 (p40/p70) expression was further analysed by flow cytometry. DCs incubated with either media alone or infected with GFP-expressing *Brucella* or *Salmonella* were labelled at 4 and 24 h post-inoculation with anti-CD11c and IL-12 (p40/p70) antibodies. Analysis of IL-12 expression was carried out on CD11c^+^ cells (Figure 5B). IL-12 expression in *Salmonella*-infected DCs peaked after 4 h, similarly to what observed with E. coli LPS (data not shown), whereas in *Brucella-*infected DCs, IL-12 expression was only detected after 24 h (Figure 5B). Similar results were obtained when analysing GFP-positive populations for each pathogen (data not shown).

These results indicate that *Brucella*, like *Francisella,* is capable of interfering with the ability of DCs to secrete cytokines. Interestingly, *Brucella* also reduced the capacity of DCs to present exogenous ovalbumin to specific T cells in the context of MHC class I and class II (Figure 5C) suggesting that *Brucella* is generally down-modulating the immuno-stimulatory properties of maturing DCs both at the cytokine production level and also at the antigen processing and presentation level.

### Identification of a *Brucella* Protein Involved in the Control of DC Maturation

**Figure 6:**
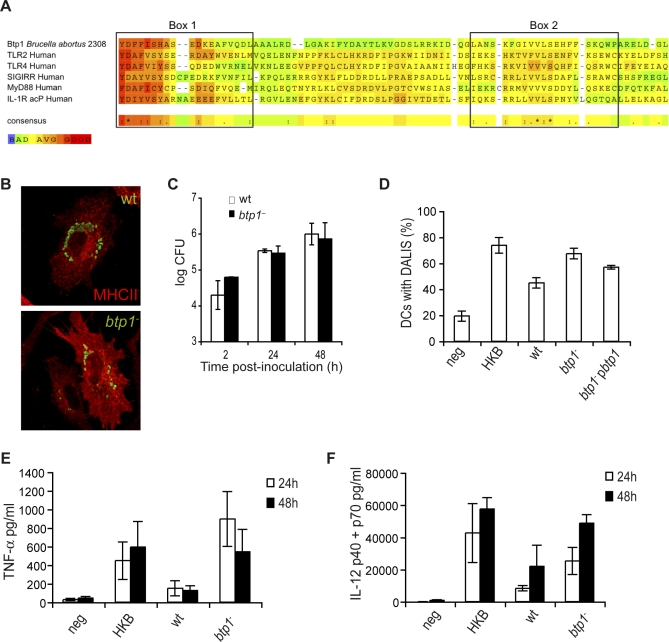
Btp1 Reduces DC Maturation (A) Identification of Btp1 as bacterial member of TLR/IL-1R (TIR) family. Comparison of the predicted amino acid sequences of Btp1 with the human members of the TIR family: TLR2, TLR4, Single Immunoglobulin IL1-R related molecule (SIGIRR), Myeloid Differentiation protein 88 (MyD88), and IL-1 Receptor accessory protein (IL-1R acP). Significant sequence similarity is observed in Box 1 and Box 2, which are important signatures of all TIR domain proteins. The alignment was constructed with T-Coffee::advanced server from EMBnet (http://www.ch.embnet.org/). The color scheme is represented in the figure and is indicative of the reliability of the alignment, with red corresponding to highest probability of correct alignment. DCs infected with wild-type B. abortus or *btp1* ^−^ were either (B) fixed at 24 h and immunolabelled for MHC II (red) and *Brucella* (green) or (C) cells were lysed at 2, 24, and 48 h and CFU enumerated. (D) Quantification of the percentage of DCs containing DALIS. DCs were incubated for 24 h with media (negative), heat-killed B. abortus (HKB), wild-type B. abortus (wt), *btp1* ^−^ mutant strain or a *btp1* ^−^ mutant with a plasmid containing *btp1* (*btp1* ^−^p*btp1*). There is a very significant statistical difference between the wt and *btp1* ^−^ (*p* = 0.0002) but only a small difference between *btp1* ^−^ and *btp1* ^−^p*btp1* (*p* = 0.05). (E and F) Analysis of TNF-α and IL-12 (p40/p70) secretion measured by ELISA from the supernatant of DCs 24 and 48 h after inoculation. All the results correspond to the means ± standard errors of four independent experiments. A statistical difference was observed between wild-type *Brucella* and *btp1* mutant at 24 h for TNF (*p* = 0.029) and IL12 (*p* = 0.045).

The *Brucella* genome was searched for candidate proteins responsible for interfering with DC maturation. We identified one candidate protein: BAB1_0279 (YP_413753), a 250-amino acid protein containing a c-terminal 130-amino acid domain with significant sequence similarity to Toll/interleukin 1 receptor (TIR) domain family (smart00255, e-value of 4E^−11^). Sequence comparison with the human TIR containing proteins TLR2, TLR4, SIGIRR, MyD88 and IL-1R acP indicated similarity particularly in box 1, the signature sequence of the TLR family, and in box 2 which is reported to be important for signaling [22] (Figure 6A). This *Brucella* protein, which we have designated Btp1 (***B*** *rucella*-**T**ir-**P**rotein 1), is also homologous to TlpA, a *Salmonella* effector that interferes with TLR signaling *in vitro* [23]. The *btp1* gene is located in a 20-Kb genomic island on chromosome I and is present in B. abortus and B. melitensis but absent from B. suis. Interestingly, the island appears to have been acquired recently via a phage-mediated integration event [24].

In order to test if this protein could be responsible for *Brucella* immunomodulatory properties, we infected DCs with *Brucella* knock-out mutants of *btp1*. We first observed that the mutant strain replicated as efficiently as the wild type *Brucella* in DCs (Figure 6B and 6C). Next, the degree of DC activation after infection with this mutant was monitored by flow cytometry and microscopy. Interestingly, the level of surface expression of MHC II and CD80 were augmented in DCs infected with the *btp1* mutant when compared the wild-type. However, CD40 and CD86 were not significantly affected (Figure S4). In contrast, a significantly higher percentage of DCs infected with *btp1* ^−^ contained DALIS at 24 h after infection (*p* < 0.001), in comparison to DCs infected with the wild-type strain (Figure 6D). Although, *btp1* ^−^ did not induce formation of DALIS to the same level than *Salmonella* (Figure 3B), it was equivalent to heat-killed B. abortus (HKB, Figure 6D) and could be partially complemented by expressing *btp1* (*btp1* ^−^ p*btp1*, Figure 6D). Therefore, the ability of *Brucella* to inhibit formation of DALIS is dependent on Btp1. In order to exclude a potential role of Btp1 on LPS immunostimulation properties, LPS extracted from *Brucella* wt and the Btp1 mutant were tested on DCs. Neither LPS from wt or *btp1* mutant was found to promote DC activation (Figure S5A and S5B). Therefore, the increase of maturation in infected cells with the *btp1* mutant is not due to modification of its LPS. In addition, an increase in the level of expression and secretion of TNF-α and to a lesser extent IL-12 (Figure 6E and 6F) was observed at both 24 and 48 h after infection, consistent with a role for Btp1 in controlling DC function. However, the IL-12 levels were lower than the levels obtained with heat-killed B. abortus suggesting that other *Brucella* factors might block DC activation. Complementation of the mutant phenotype was also attained for both TNF-α and IL-12 (Figure S5C).

### *Brucella* Btp1 Protein Modulates TLR Signalling

**Figure 7:**
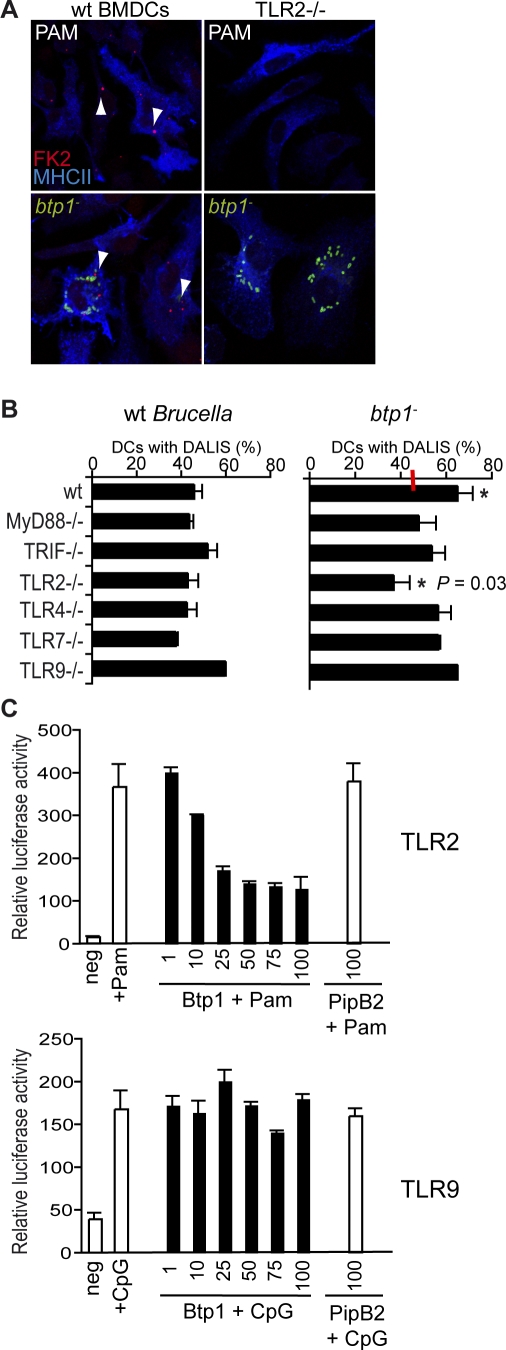
B. abortus Protein Btp1 Modulates TLR Signaling (A) Representative images obtained by immunofluorescence microscopy of DCs from wild-type mice or TLR2 knockout mice labelled for MHC II (blue), DALIS (red), and in the case of lower panel also labelled for *Brucella* (green). Cell were either stimulated with PAM (500 ng/μl, upper panel) or infected with the *Brucella* wild-type and *btp1* ^−^ mutant strains (lower panel). (B) DCs obtained from wild-type mice or different knockout mice were infected for 24 h with either B. abortus wild-type or *btp1* ^−^ mutant strain. The percentage of DALIS in infected cells was then quantified and the data represent means ± standard errors of four independent experiments. The average value for the wild-type *Brucella* is indicated with a red line. For *btp1* ^−^ we observed a statistical difference (*) between the wt and TLR2−/− DCs (*p* = 0.031). (C) HEK293 cells were transiently transfected for 24 h with three vectors: a luciferase reporter vector and either empty vector, myc-Btp1 or myc-PipB2 along with either TLR2 (upper panel) or TLR9 (lower panel). The amounts of Btp plasmids are indicated in the graph (ng). Data represent the means ± standard errors of relative luciferase activity obtained from triplicates of a representative experiment. In the case of TLR2, cells were stimulated with PAM (500 ng/μl) and for TLR9 with CpG (1 μM) for 6 h before measuring the luciferase activity.

TLR signal transduction pathways are essential for the induction of DC maturation and secretion of pro-inflammatory cytokines. The most important TLRs in the context of a bacterial infection are TLR2, TLR4, TLR5 and TLR9 that recognize lipoproteins, LPS, flagellin and CpG DNA, respectively. However, recent work showed that DCs poorly respond to flagellin due to limited TLR5 expression in these cells [25] so we therefore excluded this pathway. To determine if Btp1 was interfering with a specific TLR in the context of an infection we used DALIS formation as a read-out, since the increased number of DCs with DALIS was the clearest phenotype for the *btp1* mutant when compared to wild-type *Brucella*. DCs from different TLR knockout mice were infected with *Brucella* wild-type or the *btp1* mutant and analysed by confocal immunofluorescence microscopy. We observed that DCs from wt mice infected with *btp1* ^−^ contained a very high number of DALIS, contrasting with infected TLR2−/− DCs (Figure 7A), a phenotype reminiscent of DCs treated with the TLR2 ligand PAM. This difference was not noticeable in other TLR knockout mice used. Indeed, quantification of the number of infected DCs containing DALIS confirmed that DCs infected with the *btp1* mutant showed increased DALIS formation in wild-type, TRIF−/−, TLR4−/−, TLR7−/− and TLR9−/− DCs but not in TLR2−/− and to a lesser extent in MyD88−/− DCs (Figure 7B). These results suggest that inhibition of DC maturation by Btp1 is at least in part dependent on the TLR2 pathway. Appropriate ligands were used as positive controls for the assay (Figure S6). In addition, we found that DC maturation induced by heat-killed *Brucella*, as measured by DALIS formation, was also dependent on TLR2 (Figure S7). This result is consistent with previous reports showing that TNF-α secretion induced by heat-killed *Brucella* in DCs is partially dependent on TLR2 and MyD88 pathways [26]. We then investigated if the ability of *Brucella* to reduce DC maturation was dependent on the TLR2 pathway. DCs were infected with wild-type B. abortus for 24 h to allow establishment of a replication niche and then incubated with either LPS or PAM for a further 24 h. We found that DCs infected with wild-type *Brucella* and treated with E. coli LPS were very activated and contained very large DALIS, similar to what has been observed in LPS-treated cells. In contrast, infected cells treated with PAM contained only small DALIS similar to untreated infected cells. However, non-infected cells treated with PAM contained large and numerous DALIS (Figure S8). These results confirm that *Brucella* is able to control TLR2-dependent DC maturation.

To determine if Btp1 had the ability to specifically block TLR2 signalling we expressed increasing amounts of myc-tagged Btp1 in HEK293T cells along with a constant amount of either TLR2 or TLR9 (as a negative control) and a luciferase NF-κB reporter plasmid. We then analysed luciferase activity in the presence of the appropriate ligands. A *Salmonella* effector protein , myc-PipB2, was also included as a negative control as it is known to affect kinesin recruitment rather than cell signalling [27]. We found that Btp1 efficiently inhibited TLR2 signalling, in a dose dependent manner but not TLR9 (Figure 7C).

In conclusion, Btp1 is able to interfere with the TLR2 to reduce the progression of maturation in infected DCs.

## Discussion

*Brucella* enteric infection has been found to occur in calves' Peyer's patches [8]. Using a murine intestinal loop model, we found that after penetrating the epithelium, B. abortus localized in DCs just below the FAE of Peyer's patches. Interestingly, a few bacteria were also observed in the inter-follicular region of Peyer's patches or in the lamina propria of adjacent villi, were they were always associated with DCs. Unlike most pathogens, *Brucella* was able to establish a replication niche within DCs cultured *in vitro* in a very similar manner to what has been described in macrophages and non-phagocytic cells. DCs therefore constitute a potentially important cellular target for *Brucella* infection.

Interestingly, we have found that although the VirB type IV secretion system is required for virulence in DCs, the *Brucella* cyclic β-1,2-glucan is dispensable for early events of BCV biogenesis. The *Brucella* cyclic β-1,2-glucan, which modulates lipid microdomain organisation [15], is essential for preventing fusion between BCVs and lysosomes in macrophages and non-phagocytic cells. It is possible that within DCs early BCVs have a different membrane composition than in macrophages. In the case of human monocyte-derived DCs, *Brucella* strongly induces the formation of veils on the plasma membrane during internalisation, a phenomenon not observed in macrophages [10]. Alternatively, the proteolytic and bactericide activity of DC endosomes and phagosomes, which has been shown to be considerably limited when compared to macrophages [28,29], potentially eliminates the need for cyclic β-1,2-glucan during infection. Further work will be required to characterise in detail the molecular mechanisms of *Brucella* internalisation by DCs.

An important aspect of *Brucella* pathogenesis is its ability to evade the immune system and persist within the host. In vitro studies have illustrated that *Brucella* is efficient at remaining unnoticed by host cells, notably by having an atypical LPS [30], which is several hundred times less toxic than *Salmonella* or E. coli LPS. Although, shown to signal through the TLR4 pathway, *Brucella* LPS is not a potent inducer of pro-inflammatory cytokines and anti-microbial proteins such as the IFN-γ inducible p-47 GTPases [31]. DCs treated with up to 10 μg/ml of purified *Brucella* LPS, did not induce phenotypic maturation nor significant secretion of pro-inflammatory cytokines (Figure S4A and data not shown). Conversely, DCs incubated with heat-killed B. abortus showed significant maturation indicating that host cells can detect *Brucella* pathogen associated molecular patterns (PAMPs), other than LPS. A recent study has shown that a lumazine synthase from *Brucella* can activate DCs via TLR4 [32]. In addition, heat-killed *Brucella* can induce TLR9 and promote Th1-mediated responses in both DCs and macrophages [33] probably after bacterial degradation in lysosomes.

Immunity against *Brucella* requires cell-mediated mechanisms that result in the production of cytokines such as IL-12 and IFN-γ. In this study, we have shown that *Brucella* inhibits or delays the process of DC maturation to establish a replicative niche, which results in reduced cytokine secretion and disabled antigen presentation. It is likely that impairement of DC function and cytokine secretion by *Brucella* favours infection and/or promotes the establishment of the chronic phase of the disease. Although, we did not detect any significant secretion of IL-10 in infected DCs it is possible that, by preventing their full activation, *Brucella* uses the tolerogenic properties of DCs to subvert the immune response. There is growing evidence that induction of tolerance is not restricted to immature DCs. Recent studies have shown that migrating DCs, which are mature or in the process of maturing (bearing considerable surface expression of MHC class II molecules) are capable of inducing tolerance [34–36]. *Brucella-*infected DCs, which show an intermediate level of maturation could therefore contribute to tolerance induction in tissues and establishment of chronic infection. Consistent with this hypothesis, reduction of IL-10 levels in mice has been shown to improve host resistance to *Brucella* infection [37]. In addition, a new *Brucella* virulence protein (PrpA) was recently identified as a potent B-cell mitogen and IL-10 inducer [38]. Indeed, this protein is necessary for the early immuno-supression observed in *Brucella*-infected mice and the establishment of chronic disease. Brucellosis development is a complex process that involves many virulence factors some of them, such as the VirB type IV secretion system, are required for virulence in different cell types, whereas others may function in specific cell types, such as the *Brucella* cyclic β-1,2-glucan in macrophages and PrpA in B-cells.

A recent study has shown that *Brucella*-infected human myeloid DCs do not mature extensively and instead secrete low levels of IL-12 and TNF-α and have an impaired capacity to present antigens to naïve T cells [39]. These results are consistent with our data in murine DCs. However, this study implicates the *Brucella* outer membrane protein Omp25 and the two-component regulatory system BvrRS in the control of DC maturation through blockage of TNF-α secretion. This is not surprising as both these mutants have significant modifications of their outer membrane and LPS structure, which are an essential feature of *Brucella* virulence enabling bacteria to escape pathogen recognition [40,41]. Therefore the effects observed are more likely to be pleiotropic than specific to DC maturation. Furthermore, in the case of the *bvrR* mutant, which they show induces higher maturation than the *omp25* mutant, the increased maturation is most likely due to its degradation in lysosomal compartments (the equivalent results can be obtained with heat-killed *Brucella*) since this mutant was previously shown to be unable to replicate within human DCs by the same group [10].

In this study, we have identified a new *Brucella* protein, Btp1, which contributes to modulation of TLR signalling within host cells. A mutant lacking this protein showed increased levels of DALIS formation and also an increase in secretion of pro-inflammatory cytokines. However, the level of surface expression of some co-stimulatory molecules was not significantly increased in cells infected with the mutant strain. Thus the existence of other bacterial molecules, capable of further inhibiting the antigen presentation capability of DCs, is highly probable and additional studies are required to identify and characterize these putative new factors.

By ectopically expressing Btp1, we found that it can inhibit TLR signalling, particularly TLR2, in a manner consistent with observations in DCs obtained from different knock-out mice infected with the *btp1* mutant strain. Interestingly, we did not observe any inhibition of human TLR2 when using the same concentrations of Btp1. It is possible that higher amounts of Btp1 are required to block human TLR2 in this system or it may be that Btp1 specifically recognizes the mouse TLR2 Tir domain. Further work is now required to identify the cellular target of Btp1 and to analyse its role during infection *in vivo*. Previous studies have highlighted the importance of TLR signalling in the control of *Brucella* infection, namely the role of the MyD88 adaptor protein in the clearance of the S19 *Brucella* vaccine strain in macrophages at late stages of the infection [42]. It is interesting that *Brucella* produces at least one protein that can act as negative regulator of TLR signalling, which constitutes an essential link between the innate and adaptive immune systems. Along with the *Salmonella* TIR-containing protein TlpA, they may constitute a novel class of intracellular bacterial virulence factors that interfere with TLR signalling and control specific steps of the host immune response. We hypothesize that Btp1 is secreted into the host cytosol were it interacts with either the TLR2 directly and/or with its adaptor proteins resulting in reduction of TLR2 signalling.

In the case of *Salmonella*, TlpA is required for virulence in macrophages and in the mouse model of infection. This is not the case for Btp1, since a *btp1* mutant is not significantly attenuated in the mouse model of brucellosis (Marchesini, Comerci and Ugalde, unpublished results). Interestingly, *Salmonella* infection results in phenotypic activation of DCs and high secretion of pro-inflammatory cytokines probably due to its LPS (since addition of *Salmonella* LPS at 100 ng/ml to DCs results in DC activation; data not shown). Nonetheless, live *Salmonella* is able to restrict MHC class II-dependent antigen presentation [6], however no study has yet been carried out with the *tlpA* mutant in this context. It is possible that the Tir-containing protein TlpA is involved in the control of DC function but no mechanism has yet been proposed and it remains unclear how *Salmonella* interferes with antigen presentation in DCs. When comparing *Salmonella* and *Brucella*, it is important to consider that these two pathogens cause very distinct diseases in susceptible mice; S. typhimurium infection is characterised by fast systemic spread of the bacteria whereas B. abortus establishes a chronic-like non-fatal disease. It is therefore likely that DCs play a very distinct role in the pathogenesis of these two bacteria and that these pathogens have developed specific mechanisms to control the immune response. For example, in the case of *Salmonella*, activation of DCs does not affect its intracellular survival (data not shown) whereas *Brucella* cannot reach its ER-derived replication niche in fully matured DCs, which impact on its survival. In the case of *Brucella*, infected DCs remain in an intermediate maturation stage. Although its atypical LPS enables bacteria to remain less noticeable by the host, it is interesting that *Brucella* expresses at least one protein that interferes to a certain level with DC maturation and particularly with secretion of pro-inflammatory cytokines by DCs. Therefore, it is possible that Btp1 contributes to the establishment of chronic brucellosis by controlling in the host immune response within specific tissues. Overall*, Brucella* infection of DCs may therefore be required to control the anti-bacterial immune response, in addition to providing a productive replication niche. Alternatively, the migration properties of DCs could facilitate infection spreading, as suggested by the rapid interaction of DCs with FAE penetrating *Brucella*.

## Materials and Methods

### Bacterial strains.

The bacterial strains used in this study were S. enterica serovar Typhimurium strain 12023, smooth virulent B. abortus strain 2308 [11] and the isogenic mutants *virB9* ^−^ [43], *cgs* ^−^ BvI129 [15] and *btp1* ^−^ (this study, see bellow). In the case of *Brucella*, green fluorescent protein (GFP)-expressing derivatives contain a pBBR1MCS-2 derivative expressing the *gfp-mut3* gene under the control of the lack promoter. The plasmid pVFP25.1 carrying the *gfp-mut3* under the control of a constitutive promoter was used for *Salmonella. Brucella* strains were grown in tryptic soy broth (TSB; Sigma-Aldrich) and *Salmonella* in Luria Bertani (LB) medium. For infection, we inoculated 2 ml of media for 16 h at 37 °C up to an optical density (OD_600nm_) of approximately 2.0 [12]. *Salmonella* strains were cultured 16 h at 37 °C with aeration to obtain stationary phase cultures.

### Construction of btp1 mutant.

A PCR product of 1.257 Kbp containing the *btp1* gene (BAB1_0279) was amplified using primers 5′-caaaactctttcccgcatgcga-3′ and 5′-tcagataagggaatgcagttct-3′ and ligated to pGem-T-Easy vector (Promega) to generate pGem-T-*btp1*. The plasmid was linearized with *Eco*RV. Linearized pGem-T-*btp1* was ligated to a 0.7 Kpb *Sma*I fragment containing a gentamicin resistance cassette to generate pGem-T-*btp1*::Gm^r^. Plasmids were electroporated into B. abortus S2308 where they are incapable of autonomous replication. Homologous recombination events were selected using gentamicin and carbenicillin sensitivity. PCR analyses showed that the *btp1* wild-type gene was replaced by the disrupted one. The mutant strain obtained was called *B. abortus btp1*::Gm^r^ (*btp1* ^−^ *).*

### Construction of the btp1 complemented strain.

A 1.257 Kpb *Eco*RI fragment containing the *btp1* gene was excised from pGem-T*btp1* and ligated to the *Eco*RI site of pBBR1-MCS4 [44]. The resulting plasmid was conjugated into *btp1* mutant strain by biparental mating.

### Intestinal loop model of infection.

C57/BL/6 and BALB/c mice were starved 24h before anaesthesia. After a small incision through the abdominal wall was done, a loop starting from the ileoocaecal junction and containing 2 to 3 Peyer's patches was formed taking care to maintain blood supply. Before closing the loop, 200μL of a 10^8^ CFU/mL culture of B. abortus expressing GFP was injected. The intestine was then returned to the abdominal cavity for one hour before the mice were killed and the intestinal loops were removed, opened flat and washed extensively with PBS. Peyer's patches were then fixed with 3.2% paraformaldehyde for 60 minutes, rinsed in PBS, infused overnight in 35% sucrose and frozen in OCT compound. Immunofluorescence labelling was performed on 8 to 10 μm thick cryostat tissue sections overnight at 4°C using hamster anti CD11c (N418) and rabbit anti Brucella abortus LPS followed by incubation with goat anti-rabbit Alexa Fluor 546 and goat anti-hamster Cy5 for 1h at room temperature.

###  Bacterial infection and replication assays.

BMDCs were prepared from 7–8 week-old female C57BL/6 mice or TLR2−/− [45], TLR4−/− [46], MyD88−/− [47], TLR9−/− [48], TRIF−/− [49], TLR7−/− [50] knockout mice, as previously described [51]. Infections were performed at a multiplicity of infection of 50:1 for flow cytometry experiments and 20:1 for all remaining experiments. Bacteria were centrifuged onto BMDCs at 400 g for 10 min at 4 °C and then incubated for 30 min at 37 °C with 5% CO_2_ atmosphere. Cells were gently washed twice with medium and then incubated for 1 h in medium supplemented with 100 μg/ml streptomycin to kill extracellular bacteria (or gentamicin for *Salmonella*). Thereafter, the antibiotic concentration was decreased to 20 μg/ml. Control samples were always performed by incubating cells with media only and following the exact same procedure for infection. To monitor bacterial intracellular survival, infected cells were lysed with 0.1% Triton X-100 in H_2_O and serial dilutions plated onto TSB agar to enumerated CFUs. When stated cells were previously selected based on CD11c-labeling using CD11c MicroBeads (MACS, Miltenyi Biotec) following the manufacture's instructions.

### Antibodies and reagents.

The primary antibodies used for immunofluorescence microscopy were: cow anti-B. abortus polyclonal antibody; hamster anti-CD11c (N418; Biolegend); affinity purified rabbit Rivoli antibody against murine I-A [20]; rat anti-mouse LAMP1 ID4B (Developmental Studies Hybridoma Bank, National Institute of Child Health and Human Development, University of Iowa); mouse antibody FK2 (Biomol); mouse anti-KDEL (Stressgen). Monoclonal anti-calnexin antibody was kindly provided by Dr. D. Williams (University of Toronto). For flow cytometry allophycocyanin conjugated-anti-CD11c antibody (HL3) was used in all experiments along with either phycoerythrin-conjugated CD40, CD80, CD86, IA-IE (MHC class II) or H2-2Kb (MHC class I) all from Pharmingen. Appropriate isotype antibodies were used as controls (data not shown). For intracellular labelling of IL12 the phycoerythrin-conjugated IL-12 (p40/p70) monoclonal from Pharmingen was used.

The following TLR ligands from Invivogen were used: ODN1826, Poly(I:C), Pam_2_CSK4 and E. coli K12 LPS.

### Immunofluorescence microscopy and flow cytometry.

Cells were fixed in 3% paraformaldehyde, pH 7.4, at 37 °C for 15 min and then processed for immunofluorescence labelling as previously described [11]. Samples were either examined on a Leica DMRBE epifluorescence microscope or a Zeiss LSM 510 laser scanning confocal microscope for image acquisition. Images of 1024 × 1024 pixels were then assembled using Adobe Photoshop 7.0. In all experiments we used an antibody against a conserved cytoplasmic epitope found on MHC-II I-A ß subunits since it strongly labels BMDCs [20] while no significant labelling was detected in bone marrow-derived macrophages (Figure S2). All BMDCs significantly expressing MHC II were also labelled with an anti-CD11c antibody confirming that they are indeed DCs (Figure S2). Quantification was always done by counting at least 100 cells in 4 independent experiments, for a total of at least 400 host cells analysed.

For flow cytometry, infected DCs were collected and stained immediately before fixation. Isotype controls were included as well as DCs infected with non-gfp B. abortus as control for autofluorescence. Cells were always gated on CD11c for analysis and at least 100,000 events were collected to obtained a minimum of 10,000 CD11c-positive and GFP-positive events for analysis. A FACScalibur cytometer (Becton Dickinson) was used and data was analysed using FlowJo software (Tree Star).

### Electron microscopy.

The detection of glucose-6-phosphatase activity was performed by electron microscopy cytochemistry as previously described [52] with minor modifications. Cells were pre-fixed in 1.25% (vol/vol) gluteraldehyde in 0.1 M Pipes, pH 7.0, containing 5% (wt/vol) sucrose for 30 min on ice, washed three times for 3 min in 0.1 M Pipes, pH 7.0, containing 10% (wt/vol) sucrose and then briefly in 0.08 M Tris-maleate buffer, pH 6.5. Cells were incubated in 0.06 M glucose-6-phosphate (Sigma-Aldrich), 0.1% lead nitrate (wt/vol) in 0.08 M Tris-maleate buffer, pH 6.5 for 2 h at 37 °C. After 3 washes in 0.08 M Tris-maleate buffer and 3 washes in 0.1 M cacodylate buffer, pH 7.2, containing 0.1 M sucrose, 5 mM CaCl_2_ and 5 mM MgCl_2_, cells were post-fixed in 1.25% (vol/vol) gluteraldehyde in the same buffer for 1 h at 4 °C, and the, with 1% OsO_4_ in the same buffer devoid of sucrose for 1 h at room temperature. Samples were processed as previously described [52].

For immunoelectron microscopy samples were fixed in 4% paraformaldehyde in 0.1 M cacodylate buffer, pH 7.2, containing 0.1 M sucrose, 5 mM CaCl_2_ and 5 mM MgCl_2_, for 1 h at room temperature followed by 8% paraformaldehyde in the same buffer overnight at 4 °C. Cells were scraped, pelleted, and embedded in 10% bovine skin gelatin in phosphate Sorensen 0.1 M. Fragments of the pellet were infiltrated overnight with 2.3 M sucrose in PBS at 4 °C, mounted on aluminium studs and frozen in liquid nitrogen. Sectioning was done at −110 °C in an Ultracut cryo-microtome (Leica). The 60 nm thick sections were collected in 1:1 mixture of 2.3 M sucrose and 2% methyl cellulose, transferred onto Formvar-carbon-coated nickel grids and incubated for 2 min with PBS-glycine 50 mM and then for 20 min with PBS-1% BSA. Sections were then incubated for 50 min with primary antibody in PBS-1% BSA, washed 5 times and then labelled with 10 nm protein A-gold particles in PBS-1% BSA for 20 min. Sections were finally washed 10 times in PBS, fixed for 5 min with 1% gluteraldehyde in PBS, rinsed in distilled water and incubated for 5 min with uranyl oxalate solution. Grids were then rinsed in distilled water, incubated with 2% methyl cellulose (Sigma-Aldrich) and 0.4% uranyl acetate for 5 min on ice and then dried at room temperature. Samples were analysed with a Zeiss 912 electron microscope and images were then processed using Adobe Photoshop 7.0.

### Cytotoxicity and cell viability assays.

Measurement of lactate dehydrogenase (LDH) release in the supernatant of cells infected with different strains was carried out using the Detection Kit (Roche) as indicated by the manufacturer. The percentage of cytotoxicity corresponds to the ratio between the experimental value subtracted by the negative control (spontaneous LDH release) and the maximum LDH release (triton lysed cells) subtracted by the negative control. For detection of 7-AAD, cells were infected with GFP-expressing strains as described above and collected at 24 h in cold PBS. Cells were then labelled first for CD11c-APC, washed several times in cold PBS and then incubated with 7-AAD following the manufacturer's instructions (BD Pharmingen). The flow cytometric analysis were performed on fixed cells within 20 min.

### Cytokine measurement.

Sandwich enzyme-linked immunosorbent assays (ELISA) from Abcys were used to detect IL-12 (p40/p70), TNFα and IL-6 from supernatants of BMDCs infected with different *Brucella* strains. The ELISA kit for IFNβ measurement was obtained from PBL Biomedical Laboratories.

### Antigen presentation assay.

DCs were seeded at 1 × 10^5^ cells per well in 96-well flat bottom plates and infected for 30 min as described above. Cells were then washed and ovabulmin (Sigma-Aldrich) was added at a final concentration of 50 μg/ml for 2 h in media containing 100 μg/ml of streptomycin to kill extracellular bacteria. Antigen was then removed by changing media and DCs were co-cultured with splenic CD4^+^ T cells prepared from OTI and OTII transgenic mice (added at a 1:1 ratio) for a remaining 48 h in media containing 20 μg/ml of streptomycin. Supernantants were then collected and IL-2 production was assessed by evaluating the growth of IL-2-dependent CTLL-2 cell line (10^4^ cells/well) after 24 h and incorporation of ^3^H-thymidine (NEN, 0.5 μCi per well) during 8 h of incubation. All experiments were done in triplicates and the data presented corresponds to a representative experiment of three independent repeats. Data are expressed as the arithmetic mean counts per minute. *Brucella* did not induce cytotoxicity of DCs (Figure S2) and did not affect uptake of fluorescently labelled OVA (data not shown).

### Luciferase activity assay.

HEK 293T cells were transiently tranfected using Fugene (Roche) for 24 h, according to manufacturer's instructions, for a total of 0.4 μg of DNA consisting of 50 ng TLR plasmids, 200 ng of pBIIXLuc reporter plasmid, 5 ng of control Renilla luciferase (pRL-null, Promega) and indicated amounts of Btp expression vectors. The total amount of DNA was kept constant by adding empty vector. Were indicated, cells were treated with E. coli LPS (1 μg/ml), Pam_2_CSK4 (1 ng/μl) or CpG ODN1826 (10 μM) for 6 h and then cells were lysed and luciferase activity measured using Dual-Glo Luciferase Assay System (Promega). The TLR2 construct was obtained by PCR amplification from cDNA of bone marrow-derived macrophages and subcloning in the pCDNA3.1 expression vector (Promega).

### Statistical analysis.

All experiments were carried out at least 3 independent times and all the results correspond to the means ± standard errors. Statistical analysis was done using two-tailed unpaired Student's *t* test and *p* ≥ 0.05 were not considered significant.

## Supporting Information

Figure S1 Analysis of DC Viability during Infection (A) BMDCs were infected with either S. typhimurium, B. abortus wild-type, or *btp1* mutant strains and the supernatants collected at 24 or 48 h for detection of lactate dehydrogenase (LDH) activity. Staurosporin was used as a positive control at a final concentration of 200 nM. Results correspond to percentage cytotoxicity in relation to the negative and maximum values of LDH release and were obtained from three independent experiments. (B and C) DCs were infected with either S. typhimurium or B. abortus wild-type strains expressing GFP and analysed by flow cytometry for labelling with the vital dye 7-Amino-actinomycin (7-AAD). Results correspond to CD11c-positive (B) or CD11c GFP-positive (C) cells. The percentage of cells that are 7AAD-positive are indicated in each histogram.
(543 KB TIF)

Figure S2 Confocal Microscopy Analysis of MHC II Labelling in Bone Marrow-derived DCs and Macrophages (A) Cells were labelled with anti-MHC II (rivoli, red) and anti-CD11c (blue) antibodies and, in the case of *Brucella* (wt) with anti-LPS antibody (green). All cells showing significant MHC II labelling were CD11c-positive.
(B) Control labelling of bone marrow-derived macrophages infected with B. abortus stained with the anti-MHC II antibody (red). No significant labelling was observed in macrophages whereas some small DALIS (labelled with the FK2 antibody, blue) were occasionally observed.
(2.1 MB TIF)

Figure S3 B. abortus Fails to Replicate in Mature DCs Intracellular CFUs were enumerated by lysing cells at 2, 24, or 48 h after infection of DCs with wild-type B. abortus, in absence (black) or presence of E. coli LPS (100 ng/ml). E. coli LPS was added at either 30 min (red), 2 h (yellow), 6 h (grey), or 12 h (white) after infection. Each infection was carried out in triplicate and values correspond to means ± standard errors. There is a statistical difference between B. abortus (48 h) and B. abortus + LPS added at 12 h (*p* = 0.002) and also added at 30 min, 2, and 6 h (*p* < 0.001). The values are representative of three independent experiments.
(115 KB TIF)

Figure S4 The *btp1* Mutant Does Not Differ from Wild-type *Brucella* with regard to Expression of Co-stimulatory Molecules DCs were either incubated with media only (blue line) or infected with B. abortus wild-type (shaded grey) or *btp1* mutant (red line) expressing GFP for 24 h. DCs were labelled with anti-CD11c conjugated with APC and anti-CD40, CD80, CD86, and MHC II antibodies conjugated with PE. Representative histograms showing the PE fluorescence (FL2) correspond to CD11c^+^ cells for the negative and CD11c^+^ GFP^+^ cells for the *Brucella* infected cells.
(232 KB TIF)

Figure S5 Analysis of the Effect of LPS Extracts on DC Maturation and Complementation of *btp1* Mutant (A and B) DCs were incubated for 24 h with crude LPS extracts from either wild-type B. abortus, the *btp1* mutant, or S. typhimurium. Samples were then labelled with the FK2 antibody (red), anti-CD11c (blue), and either anti-*Brucella* LPS or *Salmonella*-LPS antibodies. DALIS are indicated by arrows. (A) corresponds to means ± standard errors of 3 independent experiments.
(C) Analysis of TNF-α and IL-12 (p40/p70) secretion measured by ELISA from the supernatant of DCs 24 and 48 h after inoculation with wild-type (wt), *btp1* ^−^, or *btp1* ^−^p*btp1*. The results correspond to a representative of two independent experiments.
(1.0 MB TIF)

Figure S6 Analysis of the Formation of DALIS in Different Knockout Mice BMDCs obtained from wild-type mice or different knockout mice were treated with the appropriate ligand for 24 h. The percentage of cells containing DALIS was then quantified by immunofluorescence microscopy. A representative from three independent experiments is shown.
(197 KB TIF)

Figure S7 Heat-Killed *Brucella* Induction of DALIS Is Dependent on Myd88 and TLR2 DCs were incubated for 24 h with either the TLR2 ligand PAM (500 ng/μl) or heat-killed *Brucella* (HK). Samples were then labelled with the FK2 antibody (red) and anti-MHC II (green).
(A) Quantification corresponds to means ± standard deviations of two independent experiments.
(1.8 MB TIF)

Figure S8 Analysis of the Formation of DALIS in the Presence of LPS and PAM DCs were infected for 24 h with wild-type B. abortus and either incubated for a further 24 h with or without E. coli LPS or PAM. Samples were then labelled with the FK2 antibody (red) antibody.
(A) Non-infected cells treated with E. coli LPS for 24 h.
(B) Wild-type *Brucella* gfp-infected cells at 48 h post-infection.
(C) wild-type *Brucella* gfp-infected cells (24 h post-infection) further treated with E. coli LPS for another 24 h.
(D) wild-type *Brucella* gfp-infected cells (24 h post-infection) further treated with PAM for another 24 h (see on the same coverslip the upper infected cell displaying a much less maturation pattern than the lower cell that has not been infected).
(376 KB TIF)

### Accession Numbers

The Entrez Protein (http://www.ncbi.nlm.nih.gov/sites/entrez?db=protein) accession number for Btp1 (BAB1_0279) is GI: 82699179.
